# Glycyrrhizic acid aggregates seen from a synthetic surfactant perspective

**DOI:** 10.1039/d3cp04835g

**Published:** 2023-12-23

**Authors:** Peter Fischer, Viviane Lutz-Bueno

**Affiliations:** a Institute of Food, Nutrition and Health, ETH Zurich 8092 Zurich Switzerland peter.fischer@hest.ethz.ch; b Laboratory of Neutron Scattering and Imaging, Paul Scherrer Institut PSI 5232 Villigen Switzerland viviane.lutz-bueno@psi.ch

## Abstract

Bio- or plant-based surfactants are a sustainable and renewable alternative to replace synthetic chemicals for environmental, drugs and food applications. However, these “green” surfactants have unique molecular structures, and their self-assembly in water might lead to complex morphologies and unexpected properties. The micellization of saponin molecules, such as glycyrrhizic acid (GA), differs significantly from those of conventional synthetic surfactants, yet these differences are often overlooked. Saponins self-assemble in complex hierarchical helical morphologies similar to bile salts, rather than the expected globular, ellipsoidal and wormlike micelles. Here, we review two potential routes for molecular self-assembly of GA, namely kinetics of crystallization and thermodynamic equilibrium, focusing on their structure as a function of concentration. Some uncertainty remains to define which route is followed by GA self-assembly, as well as the first type of aggregate formed at low concentrations, thus we review the state-of-the-art information about GA assembly. We compare the self-assembly of GA with conventional linear surfactants, and identify their key similarities and differences, from molecular and chemical perspectives, based on the critical packing parameter (CPP) theory. We expect that this work will provide perspectives for the unclear process of GA assembly, and highlight its differences from conventional micellization.

## Abstract 2

Bio- or plant-based surfactants are a sustainable and renewable alternative to replace synthetic chemicals for environmental, drugs and food applications.

## Graphical abstract

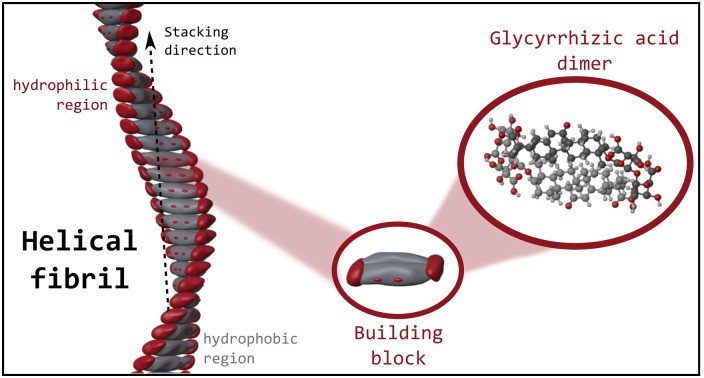

## 1 Introduction

Green surfactants are of growing interest due to the increased awareness of the negative environmental impact of conventional synthetic surfactants, which are typically derived from non-renewable resources and are often non-biodegradable, leading to long-term accumulation in the environment. In contrast, green surfactants are derived from renewable resources and are often biodegradable, making them a more sustainable and environmentally friendly alternative. Furthermore, the production and use of green surfactants may contribute to a reduction in carbon emissions, thereby helping to mitigate climate change. As a result, there is a growing demand for green surfactants in various industrial applications, ranging from cleaning products to pharmaceuticals.^1^

However, the self-assembly and morphologies that natural molecules form are much more challenging to predict than for conventional surfactants. This prediction depends on the molecular geometry, specifically on the ratio of hydrophobic and hydrophilic groups in the molecule's structure in the presence of water, which might depend on the pH, intermolecular interactions, chirality, and spatial arrangement. Different from synthetic surfactants, such as cetyltrimethylammonium bromide (CTAB), most of the plant-based molecules do not have a clear balance of hydrophobic and hydrophilic groups, and the molecular structures are unique. As a consequence, complex chemical synthesis and purification are often needed to improve the separation among hydrophobic and hydrophilic moieties in plant-based molecules, and thus enable their self-assembly.^2^ The controlled and predictive self-assembly of plant-based compounds is still rare, since theories such as critical packing parameter (CPP)^3^ are not directly applicable. Up to now, the determination of whether a green surfactant will self- or co-assemble with other molecules still relies mainly on exploratory screening as well as trial and error procedures.^4^ Greater understanding and application of such molecules require deeper characterisation and understanding of their basic adsorption and self-assembly properties under controlled conditions.^5^

From a “conventional” surfactant perspective and physico-chemical aspects, we discuss the challenges of working with plant-based green surfactants, using saponins as model systems. This work focuses solely on glycyrrhizic acid (GA), due to its complex molecular structure and its impact on the self-assembly process and morphology. We review the influence of the large molecular volume, “headgroup” size and flexibility, “tail” rigidity, and deprotonation on the self-assembly, and morphology. The discussion will then focus on the mechanisms of self-assembly, and on structural characterization mainly based on scattering techniques. Due to some discrepancies about the absolute values of characteristic dimensions, we concentrate on general trends rather than a detailed discussion of specific values. Lastly, we include a perspective for the replacement of synthetic surfactants by plant-based amphiphilic molecules in cases that self-assembly is required, and the main challenges and opportunities lying ahead in the field.

## 2 Molecular structure

**Fig. 1:**
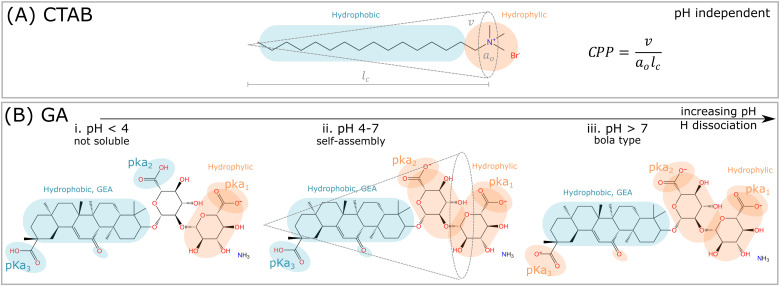
Comparison among synthetic surfactants (CTAB) and plant based molecules (GA). (A) The critical packing parameter (CPP) is defined, where *v* is the volume of the hydrophobic tail, *a*_0_ is the area of the hydrophilic headgroup, and *l*_c_ is the length of the tail. The critical packing parameter (CPP) is nearly independent of pH, and depends rather on ionic dissociation and neutralization of the bromide (Br^−^) counterion. (B) The hydrophilic and hydrophobic parts of the GA molecule depends on the pH. i. For pH < 4, the hydrogen of the carboxylic group p*K*_a1_ dissociates, and the molecule becomes nearly insoluble in water. ii. In the range 4 < pH < 7, the hydrophilic and hydrophobic parts are balanced, since both carboxylic groups p*K*_a1_ and p*K*_a2_ are dissociated. In this pH range, self-assembly might occur, and the interpretation of such process by CPP is potentially feasible. iii. For pH > 7, all hydrogens from the carboxylic groups are dissociated, and the GA molecule becomes bola-type, which is too repulsive to self-assemble.

Glycyrrhizic acid (GA) is one example of a surfactant molecule that can be directly extracted from a plant, being an interesting food-grade natural surfactant.^6^ GA also has an amphiphilic structure, similarly to synthetic surfactants, such as CTAB (Fig. 1A). Apart from the L-shape of the GA molecule shown in Fig. 1B, another relevant distinction to CTAB is that the ionization of the carboxylic acid groups depends on pH, which will directly impact the definition of headgroup and tail, and consequently, the critical packing parameter (CPP) and the type of morphology formed by the GA molecule. At high pH, GA has the potential to become a bola-type surfactant (Fig. 1A), which comprises a hydrophobic aglycone moiety with one carboxylic group, and a hydrophilic diglucuronic acid moyeties with two carboxylic groups at the opposite end of the hydrophobic skeleton.^6^

These dependencies are derived from the polyprotic weak acid structure of GA. Since it has three carboxylic groups, and thus, it also has three acidic dissociation constants (*K*_a_), which indicate how completely an acid dissociates in an aqueous solution. The p*K*_a_ measures the strength of an acid, *i.e.* how tightly a proton is held. The lower the value of p*K*_a_, the stronger the acid and the greater its ability to donate its protons. The p*K*_a_ values of GA, determined by potentiometry, are p*K*_a1_ = 3.98, p*K*_a2_ = 4.62 and p*K*_a3_ = 5.17 (see order in Fig. 1B).^7^ Denk *et al.*^8^ suggested that the carboxylic groups p*K*_a2_ and p*K*_a3_ have equal apparent dissociation constants of 5.01. Above pH 4, the ionization of carboxylic groups proceeds gradually until is complete at pH 8.^9^ As a consequence, the carboxylic groups contribute to the hydrophilic domain of GA molecules, but also provide negative charges upon hydrogen dissociation. Thus GA has properties of both anionic and nonionic surfactants, depending on the hydrogen dissociation of the carboxylic and hydroxyl groups at different pHs.

### Critical packing parameter

For this discussion, we divide the GA molecule into “headgroup” and “tail” portions, comparing these to conventional synthetic surfactants, such as CTAB (Fig. 1A). The theory of Israelechvili provides the conventional evolution of micellar self-assembly,^3^ in which the headgroup size (*a*_0_) and shape, maintaining the same tail chain length (*l*_c_) and chain volume (*v*), should impact the CPP, as well as the preferential curvature, and define the morphology of the self-assembled structure, according to the formula in Fig. 1A. An increase in effective area/molecule caused by larger headgroup, while maintaining the same tail size, should result in more globular micelles, since the preferred curvature increases.^10,11^ However, this geometrical interpretation faces limitations to be applied to saponins, because of their complex molecular structure and dependence on pH.

The hydrophobic “tail” of GA (Fig. 1B) is a chiral rigid skeleton, composed of the molecule glycyrrhetinic acid (GEA), which has multiple reaction sites that provide easy attachment to other functional structural units. The geometry of GEA is very different from conventional surfactants, which have flexible, linear, hydrophobic and long hydrocarbon tails (Fig. 1A). Additionally, GA molecules are responsive, due to the presence and position of the hydroxyl and carboxyl groups.^12^ The rigid tail, GEA, can be combined with a series of hydrophilic headgroups and form building blocks with diverse CPP, a concept classifying saponins. Different from CTAB that forms liquid-like micellar core composed of hydrocarbonic tails, the rigid hydrophobic skeleton provides higher thermal stability and mechanical properties, and leads to properties such as self-healing and shape memory,^13^ which are highly relevant for self-assembled functional materials. However, the same rigidity leads to limitations in packing, due to the lack in flexibility for the tails to form a compact micellar core, and generates incomplete separation of the hydrophilic and hydrophobic domains. These properties are similar to human-derived bile salts, which can self-assemble into unique aggregates because of their rigid molecular structure.^14^

In conventional surfactants, the headgroups are small, flexible and hydrophilic with or without polar and/or charged molecules. In saponins, these hydrophilic headgroups are mainly composed of saccharides. The most common saponins have between 2 and 4 saccharides attached to the GEA skeleton, which will follow different reaction sites and conformations. While GA has two saccharide groups as the headgroup, aescin has 3, and tea has 4, being the largest headgroup among them. Based on small angle neutron scattering (SANS), Tucker *et al.*^5^ suggested that saponins follow the CPP theory. Larger headgroups, such as tea saponin, form globular micelles with higher curvature, while smaller headgroups, such as aescin and GA, lead to more elongated micelles. At low temperature (10 °C), the aescin micelles are reported to be rodlike, whereas at high temperature (40 °C) the structure is ellipsoidal. These measurements were performed for aescin in phosphate buffer at pH 7.4 with concentrations of 1.7–9.5 mM. The radius of gyration is estimated by SANS to be 31 Å for rodlike micelles at 10 °C and 21 Å for ellipsoidal micelles at 40 °C.^15^ The area of the headgroup (*a*_0_) influences the CPP of synthetic charged surfactants, such as CTAB. However, this headgroup size also changes upon the penetration of hydrophobic co-surfactants (sodium salicylate, for example), and upon the neutralization with counterions (sodium chloride, for example).^16^

Apart from molecular geometry and CPP, the hydrophobicity of a molecule also determines self-assembly. The headgroup of aescin, for example, has an additional saccharide group compared to GA, however it also has one less carboxyl group, and it is then less hydrophilic. This property causes a significant impact on the internal micellar packing in the core, and both GA and aescin lead to similar elongated aggregates.^5^ Furthermore, reports on tea saponins describe the formation of spherical micelles in water, which is attributed to the large headgroup (and CPP). However, we should also highlight that the headgroup of this molecule does not contain carboxylic groups, thus its hydrophobic–hydrophilic balance is less dependent on pH. Among the mentioned saponins, GA has the smallest number of saccharide groups, but the greatest number of carboxyl groups, thus its effective headgroup area/molecule is actually larger than for aescin and tea. The presence of such carboxyl groups impacts the molecular shape, the polar-apolar balance, and water affinity of GA. Changes in pH will then lead to larger or smaller hydrophilic headgroups, and only for GA, due to the distribution of its carboxylic groups, will lead to the transition to a bola-type amphiphilic behavior. Beyond providing polar domains, the hydroxyl and carboxylic groups in the GA molecule may participate in intermolecular hydrogen bonds.^14^

Ultimately, three factors impact the headgroup and the molecular packing of GA molecules into aggregates: the number of saccharide groups, the number of carboxyl, groups and their distribution on the molecule.^17^ In this way, the CPP should be also dependent on the intra and inter-headgroup interactions between the saccharide and carboxyl groups under specific pH conditions. This dependency challenges the CPP theory by Israelechvili, and the conventional evolution of micellar size and shape,^3^ suggesting that not only the molecular geometry and size, but other factors such as hydrophobicity under specific conditions, and the presence of hydrogen bonds can impact the headgroup area and thus, the CPP of complex amphiphilic molecules.

### Surface activity of GA

When surfactants remain at interfaces, the interactions between the surfactant, oil, and water molecules determine the penetration depth of the oil molecules at oil–water interface, thus the packing of the surfactants in the adsorption layer. At non-polar oil interfaces, the oil molecules are squeezed out from the interfacial layer. On the other hand, polar oil molecules interact with the aqueous phase and the increased affinity to the interface results in a competition between the polar oil molecules and the surfactant. In such mixed adsorption layer, the minimum area per surfactant molecule increases, *i.e.*, a looser packing of the surfactants is observed.^18,19^ Similar to the penetration of counterions into the surfactant layer, the CPP is thus also influenced by the polarity of the oil, *i.e.* ability to co-adsorb at the interface. The packing at planar air–water interfaces offer different constraints to those in the self-assembly. The surface-activity of GA at the air–water interface is limited by the rigidity of the hydrophobic group, which is naturally disadvantageous, compared to the flexibility of the hydrocarbonic tail of synthetic surfactants.^9,20,21^ The pH also plays a role for surface-activity, due to the dissociation of carboxylic groups. Consider that *A* is the area occupied by a single GA molecule at the air–liquid interface. At pH 5, *A* is 1.8 nm^2^ per molecule, and at pH 6, *A* decreases to 0.57 nm^2^ per molecule.^9^ For aqueous solutions with low ionic strength, the low interface adsorption at pH 5 depends on the negatively charged carboxylic acid groups, which leads to high repulsive charges, and consequently, prevents adsorption at the interface.^22^ Larger *A* usually results in ineffective adsorption at interfaces, thus at pH 5, the GA molecules remain mainly in bulk, rather than adsorbed at the interface. At pH 6, the GA monomers are more ionized, have higher repulsion, and move from bulk solution to the interface, because their aggregation in bulk becomes disadvantageous. GA forms a single uniform layer of about 3.5 nm in the concentration regime of 0.04 to 4 mM, when added to water without a buffer solution (pH 4–5).^5^ The maximum packing at the saturation point is believed to be limited by the interactions among the hydrophilic headgroups of the molecule, while the hydrophobic skeleton plays a rather secondary role.^5^

### GA as acid or salt

In the works we cite, we carefully examined whether the GA molecule was used in its original acid form or neutralized as a salt. The aqueous solubility of neutral glycyrrhizic acid is relatively low, being below 1.5 mM for pH < 5. For buffer solutions with pH > 5, the solubility increases rapidly.^20^ For practical use, GA is typically dissolved in a buffered aqueous solution or neutralized as an ammonium or alkali salt in its crystalline form ref. 9. The ammonium ion remains close to the carboxyl group indicated in Fig. 1 by p*K*_a2_.^9^ GA mono ammonium salt dissociates as the pure acid in MilliQ water,^23^ thus the neutral and anionic forms are comparable for structural analysis.^24^ We consider the same comparativeness for the data acquired in the cited literature, and will make no differentiation between salt and acid forms in the upcoming text. To ensure no effect from the dissociated ammonia (or other ions) in the formulation, GA can be purified.^22^

## 3 How do the GA molecules self-assemble?

Increasing efforts have been made to use GA, as structural building blocks to form functional supramolecular assemblies for food, drug delivery and material's science applications, due to their multiple functional groups, rigid skeletons, and unique stacking behaviors.^25^ However, some fundamental understanding of their self-assembly behavior is lacking. An ongoing debate in literature is whether GA has a clear critical micellar concentration (CMC) and forms micelles, or if has a critical aggregation concentration (CAC), and forms dimeric building blocks that stack into fibrils. The self-assembly of GA into long fibrils is known,^23^ and it seems to follow kinetic processes rather than thermodynamic. Apart from geometric shape, there are two main processes that describe how molecules self-assemble: thermodynamic processes occur when molecules spontaneously self-assemble, due to the minimization of free energy; and kinetic processes occur when molecules self-assemble, due to their random motion and collisions, and may or may not form larger aggregates depending on the energy and orientation of these collisions. In this process, the rate of aggregation is determined by the frequency of collisions, the kinetic energy of the molecules, and their relative orientation. Here we propose that GA molecules do not form the classical micelle type, *i.e.* follow the thermodynamic processes similar to surfactant solutions. Different from polymer solutions, in which monomers are covalently bonded, surfactants self-assemble by relatively weak physical attractions. The micelles are in equilibrium, and break and reform continuously, where the kinetics of breakage and reformation reactions are greatly dependent on the surfactants and salt concentrations, temperature, and flow. The dynamic break and reform of micelles is the reason that they are also called living polymers.^26^

Up to now, different properties and morphologies have been reported for the self-assembly of GA under different experimental conditions, in particular pH, and a general model describing such process remains missing. It is known that the morphology, and the viscoelastic response of GA changes as a function of concentration, and that the structure goes from an isotropic fluid to a nematic phase composed of fibrils, and then forms a hydrogel, upon fibril entanglement. However, in literature various types of structures are mentioned and measured by small angle scattering such as dimers, globular and elipsoidal micelles, nanoclusters, ultrafine semi-flexible helical nanofibrils, nematic liquids and hydrogels. Currently, there is no consensus about the process that forms such morphologies, especially the first aggregate at the dilute regime, and the growth dependence on concentration and pH.

### 3.1 Micelles

#### CMC

Upon increasing concentration, solution of surfactant molecules first increase the monomer concentration, and then saturates around the critical micellar concentration (CMC), when they start to self-assemble into micelles. Back in 1964, pioneer conductivity and surface tension measurements did not show signs of GA micellization.^27^ Recently, the CMC of GA was measured in buffer solutions at 25 °C, and estimated to be approximately 0.25 mM at pH 5.1, and 0.38 mM at pH 5.5.^28^ Later, Matsuoka *et al.*^20^ investigated the CMC as a function of pH by surface tension measurements. It was estimated that at pH 5, the CMC is about 2.9 mM, and increases to 5.3 mM at pH 6. The solubilization of pyrene in the micellar core of GA generated fluorescent signals above the same CMC range.^20^ In another study, the CMC of GA dissolved in water without a buffer solution was estimated by neutron reflectometry to be between 0.1 mM and 1 mM.^5^ In general, CMC values were reported up to pH 6, but at a neutral pH values, GA does not show a clear CMC.^20^ The reported CMC for GA are comparable to conventional surfactants, for example CMC = 0.9 mM for CTAB,^29^ while the CMC of bile salts is at a higher range of about 4–20 mM for cholic acid.^14^

It is believed that saponins, similarly to synthetic surfactants, can self-assemble into micellar solutions, mainly driven by hydrophobic forces.^30^ However, various studies indicate the presence and/or absence of CMC, and lack in highlighting the importance of hydrogen bonds. The positions and orientations of the hydroxyl groups in GA enable these molecules to form hydrogen bonds among them. The directional and specific nature of the hydrogen bonds limits the molecular orientations, introducing additional ‘rigidity’ into such aggregates, similar to bile salts.^14^ In this case, hydrogen bonds become a complementary mechanism for self-assembly, in addition to the hydrophobic effect which has no direction nor specificity. The intricate balance between these driving mechanisms depends on the molecular shape as well as the solvent conditions, such as temperature, concentration, pH and ionic strength. It should also be clear that the CMC of GA will depend drastically on the solvent's pH, because of carboxylic groups dissociation. For bile salts, the transition concentration from monomers to micelles is broader than for conventional amphiphiles, and instead of a CMC, a “noncritical multimerization concentration” is suggested.^31^ Another suggestion is the existence of two CMCs. The first CMC is related to the formation of small aggregates (multimers), and the second to the existence of stable micelles,^32^ or fibrils, as discussed later. Here, there are opportunities for detailed studies of the morphology of GA assemblies at low concentrations.

#### pH

Fig. 1B shows an overview of the hydrophilic–hydrophobic balance changes in the GA molecule as a function of pH. At pH < 4, the carboxyl groups of GA are protonated (–COOH), and therefore the overall molecular charge decreases, being predominantly non-ionic. The GA molecule becomes almost electro-neutral for very acidic conditions around pH 2.^22^ However, the low dissociation of the carboxyl groups results also in lower solubility in water. Therefore, fully protonated forms of GA are not soluble in water, and cannot self-assemble, probably remaining in solutions as monomers or dimers.^8^ The formation of GA dimers is proposed by Zelikman *et al.*^33^

At pH > 7, most of the carboxylic acid groups are dissociated, and therefore the GA molecules is highly negatively charged (–COO^−^), thus the solubility becomes higher (Fig. 1B). Over pH 8, of all three carboxylic groups are completely dissociated, even the one in the hydrophobic portion.^20^ It causes the loss of the clear amphiphilic structure and induces repulsion effects between different GA molecules, avoiding their aggregation.^34^ As a result, complete ionization results in an increase in hydrophilicity, and the molecule changes to a bola-type surfactant having hydrophilic groups at both ends (Fig. 1B).

The dependence of micellization on pH is disadvantageous for practical use without well-controlled conditions. However, this unique behavior originates mainly from the weak acidity of the three carboxylic groups in the GA molecule, which affects the sensitive balance between hydrophobic and hydrophilic properties. The hydrogen bond between the ionized hydroxyl groups can form unique structures.^35^ As a consequence, GA molecules can self-assemble within a narrow range of pH 4–7,^20^ when carboxylic groups of GA are partially dissociated.^36^ Only then, mono- and di-deprotonated GA molecules can exist in solution, and self-assemble into complex organized hierarchical structures, such as helical fibrils.^23^

#### Ionic strength

High ionic strength can screen the electrostatic repulsion among molecules, and favor their self-assembly, while decreasing the CMC.^22^ However, the charges in the GA molecule depend on pH, thus for the ionic strength to have a controlled effect, the carboxylic groups of GA should be dissociated at pH > p*K*_a_. Monovalent ions (*e.g.*, sodium Na^+^, potassium K^+^) can mostly screen electrostatic charges, while multivalent ions (*e.g.*, calcium Ca^2+^, aluminium Al^3+^) may additionally form bonds surfaces, causing charge reversal, *i.e.* initially negatively charged molecules, such as the dissociated carboxylic groups in GA, may become positively charged by binding of ions.^22^ GA is nonionic, and becomes weakly ionic at higher pH, thus any impact of electrolyte is unexpected. At high pH, the presence of high amount of negative charges in carboxylic acid groups can attract positive ions, and bring the electrical charge towards electro-neutrality in a process that occurs faster than self-assembly. The long-range attractive interactions, including van der Waals, become stronger and start to dominate the long-range repulsive interactions.^22^ As a consequence, the addition of electrolytes to GA solutions reduces the CMC of ionic surfactants, and multivalent ions have greater impact, which was confirmed by the unaffected saturation absorption measurements with the addition of electrolytes.^5^

#### Structure

**Fig. 2:**
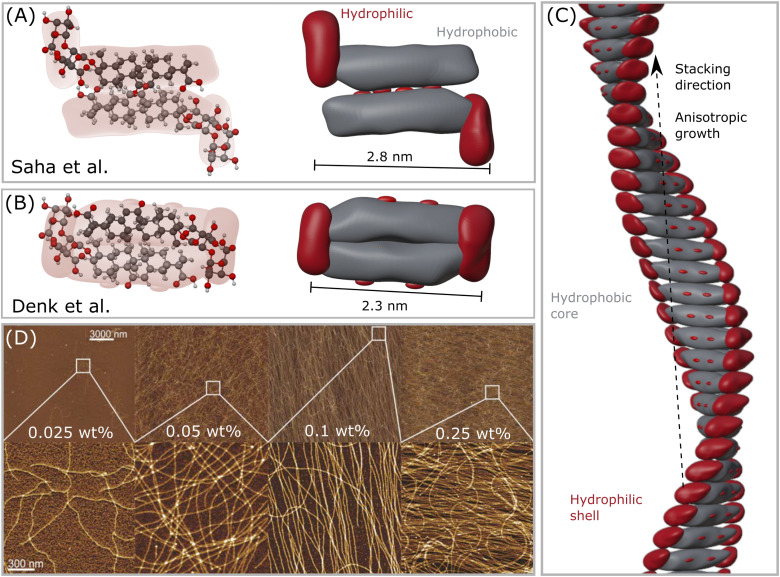
(A) The first GA dimer assembly proposed by Saha *et al.*^23^ (B) The updated interpretation of the GA dimer by Denk *et al.*,^8^ which forms a more compact building block. (C) Helical fibril assembled by stacking the dimers in (B), forming a hydrophobic core-like axis, while the hydrophilic moieties remain mostly exposed to water, forming a shell–core structure. Note that this assembly might only occur in the range 4 < pH < 7, which enables self-assembly of GA molecules. (D) Atomic force microscopy (AFM) height images of GA fibrils in water of selected areas at increasing concentrations. Reproduced from Saha *et al.*^23^ with permission from John Wiley and Sons, copyright 2015.

Pioneer small angle X-ray and neutron scattering (SAXS and SANS) and experiments indicated that GA forms rod-like micelles. Matsuoka *et al.*^20^ measured SAXS signals for two buffer conditions (pH 5 and pH 6) and covered a range of GA concentrations from 3 to 8 mM. The analysis is performed based on the Guinier approximation to a slope of −1 towards low scattering vectors *q*. By fitting the rod-like form factor, an object with an radius of 1.5 nm and length of 21 nm was found at 5 mM in pH 5. The dimensions of the object decreased to a radius of 1.3 nm and length of 18 nm at 8 mM in pH 6.^20^ This decrease indicates that the pH and concentration impact the assembly of GA molecules. Another work estimated the formation of slightly elongated micelles with aggregation number of 270, radius of 1.4 nm and length of 1.9 nm with 5 mM GA and 100 mM sodium chloride (NaCl) in water without controlled buffer conditions.^5^ In this case, the pH decreases to values around 4 upon the dissolution of GA in water. These slightly anisotropic globular micelles remained isotropic under shear flow.^5^ The GA molecule has a energy minimized molecular length of 1.86 nm. Thus, the classical micelle with a well-defined core and shell structure is unlikely to form with the reported assembly radius of about 1.5 nm. Assembly processes similar to bile salts are more likely to occur for GA in the range of pH, due to its dependency on pH and rigid structure. The rod-like micellar structures, suggested by scattering techniques, might be instead short helical fibrils, which follow the process described in Fig. 2.

### 3.2 Fibrils

When simply dissolved in water without buffer control, GA can form solutions with pH around 4, and self-assemble into long fibrils at concentrations higher than 0.3 mM.^23^ GA fibrils are prepared by dissolving GA in MilliQ water in a sealed vial, heating it at 80 °C to accelerate the dissolution with occasional shaking until a clear solution, followed by incubation of the sample for 24 hours at 25 °C. Saha *et al.*^23^ pioneered the detection of such fibrils, and measured atomic force microscopy (AFM) of dried samples on a mica substrate. Samples with 0.3 mM (0.025 wt%) of GA already showed a few very thin fibrils. From concentrations around 0.6 mM (0.05 wt%) GA, fibrils with length of tens of micrometers were found in equilibrium with a lower amount of GA monomers. At concentrations of 1.2 mM (0.1 wt%) GA, almost all of the monomers were aggregated into fibrils. At concentrations of 3 mM (0.25 wt%), the GA fibrils covered the whole surface of the mica substrate.^23^ Higher concentrations will then form a hydrogel. Some polyvalent metal ions (such as calcium Ca^2+^, copper Cu^2+^, and zinc Zn^2+^) promote the gelation of GA at concentrations lower than 24 mM. The addition of a small amount of Zn^2+^, for example, reduced the gelation concentration of GA to 6 mM (0.5 wt%).^37^ The gelation concentration of GA increases in the following order: Zn^2+^ < Cu^2+^ < Ca^2+^. A monovalent silver ion Ag^+^ did not influence the GA gelation concentration, while trivalent iron ions Fe^3+^ could even combine with GA, forming precipitates.^37^

#### Structure

Compared to the dilute systems, and to the descriptions of micellization processes, a better consensus was reached in the literature regarding the morphology of GA fibrils at concentrations above 3 mM.^8,23^ The L-shaped GA molecules are expected to assemble into dimeric building blocks (Fig. 2A and B), which then are stacked into a right-handed helix with thickness of 2.5–3 nm and periodicity of 9 nm (from AFM, SAXS and SANS data, see Fig. 2C). These fibrils have a core–shell structure, with the hydrophobic part forming the core that serves as the fibril main axis, and a shell composed of the hydrophilic headgroups and functional groups facing towards the water (Fig. 2C). It was reported that these dimensions remain similar, independently of the GA concentration, which indicates that the building blocks remain unaltered and that only the number of stacked building blocks increases with an increase in concentration,^23^ indicating that at lower concentration shorter fibrils are formed rather than rod-like micelles, as previously suggested.^20^ GA fibrils do not follow the clear concentration-induced growth of conventional synthetic surfactants,^16^ but their anisotropic growth in length depends on parameters, such as ionic strength, pH and temperature (Fig. 2D). Even though there is a clear self-assembly of GA, the structure of the GA aggregate remains unknown, especially at low concentrations. We suggest that dimers, and then short, followed by long fibrils are probably formed upon increasing concentrations. This process is supported by nuclear magnetic resonance (NMR) data, which shows that the chemical shifts of GA, and the interactions among molecules, are sensitive to changes in pH, but not sensitive to the concentration of GA,^36^ which suggests that similar structures are formed at low and high concentration. The complex interplay between the ‘unspecific’ hydrophobic effect and the ‘specific’ hydrogen binding leads to unexpected CMC values for GA. In this case, the GA self-assembly into fibrils is not dominated by hydrophilicity (or solubility), but by the hydrogen bonding.

### 3.3 Fibrillar hydrogels

The dissolution of GA molecules in water drops the pH to about 4.5, being ideal for gel formation. The formation of GA hydrogels involves two steps: (1) GA self-assembles into fibrils through hydrophobic interactions (repulsive interaction between polar and nonpolar groups) and hydrogen bonds, forming a highly organized structure; and (2) the increase of concentration fuses the fibrils into a network structure by entanglement (Fig. 2D). Such non-covalent interactions impart the hydrogels with excellent shear-thinning, self-healing ability and good viscosity.^38^ Under high shear rates, the shear viscosity of 12 mM (1 wt%) GA hydrogel is close to the magnitude of pure water. For hydrogels with 18 mM (1.5 wt%) and 24 mM (2 wt%), the shear viscosity is one order of magnitude higher than pure water.^39^ The oscillatory rheology of the hydrogel with 24 mM (2 wt%) GA is of a viscoelastic network. It was suggested that the fibrils are anisotropically ordered within nematic mesophases.^8^ Apart from pH-responsive, the GA hydrogel is also thermo-responsive with a reversible gel–sol transition at 55–60 °C. Above this temperature range, the hydrogen-bonding fibrillar network structure becomes unstable.^25,40^ Higher ionic strength reduces the electrostatic repulsion between the GA fibrils, enhancing the inter-fibrillar aggregation, the hydrogen bond interactions, and resulting in the increase in the turbidity of hydrogels.^41^

**Fig. 3:**
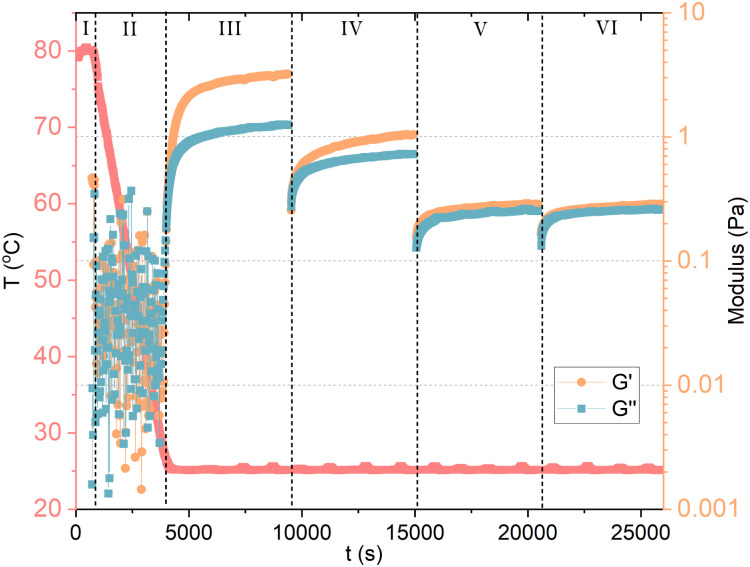
Oscillatory and shear rheology of 10 mM GA in water. In stage (I), the temperature was raised to 80 °C, while shearing the fluid to ensure dissolution. The sample was then cooled to 25 °C in stage (II) under shear. During stage (III), we measured oscillatory rheology and reached the stability of the viscoelastic properties of GA hydrogel. Between stage (III) and (IV), we imposed again high shear. Notice that shearing the sample reduces *G*′ and *G*′′ in stage (IV), which is then restored at a lower rate. Again, a high shear step was applied between stage (IV) and (V), and between (V) and (VI), which seems to have disrupted the viscoelasticity of the system within the time scale observed.

Denk *et al.*^8^ reported a important proof of the kinetic behavior of GA self-assembly: the critical gel concentration (around 24 mM) does not only depend on formulation, but also on the methodology of sample preparation. If samples are heated, and re-cooled to room temperature without agitation, a weak gel can already form at 6 mM (0.5 wt%) GA, compared to 24 mM (2 wt%) without heating. The heating during homogenization of the GA sample can affect the gelation process, leading to either turbid or clear gels. The turbidity of the gels was not assigned to a macroscopic phase separation, but rather to inhomogeneities in the gel network.^8^ Although, vigorous shaking of the sample leads to a transition from a gel to a fluid state. At rest, the gel can reform. Samples containing 6 mM (0.5 wt%) to 18 mM (1.5 wt%) of GA that are prepared without heating can flow under gravity, and are birefringent. These solutions are also capable of forming hydrogels, but they need to be heated and re-cooled with sufficiently low agitation. The cooling rate certainly influences the gelation process. Lower cooling rates favor the growth of longer fibrils, which contribute to the networks of a gel. Here, it becomes clear that the self-assembly of GA has a kinetic origin, rather than thermodynamic, and that it should be treated as a fibrillar gel. To confirm, we measured 10 mM GA hydrogel with oscillatory and shear rheology, as shown in Fig. 3. The GA and water were mixed *in situ*, within a Couette cell with a gap of 1 mm (Thermo Scientific Haake Mars III rheometer). In stage (I), the temperature was raised to 80 °C, while shearing the fluid at 100 s^−1^ to ensure dissolution for 10 minutes. The sample was then cooled to 25 °C in stage (II) during 55 minutes while the shear was maintained at 100 s^−1^. During stage (III), we measured oscillatory rheology with a frequency of 1 Hz and shear strain of 0.5 Pa for 90 minutes, until the viscoelastic properties reached stability, confirming the formation of a hydrogel. Before stage (IV), we imposed 2 min of 100 s^−1^. Notice that shearing the sample reduces the storage (*G*′) and loss (*G*′′) moduli in stage (IV), which is then restored at a lower rate during the upcoming 90 minutes of measurement. Now, we apply an even higher shear rate of 1000 s^−1^ for 2 minutes between stage (IV) and (V), and again between (V) and (VI), which seems to have disrupted the viscoelastic structure of the fibril hydrogel within the time scale observed. The recovery of the system's viscoelaticity as a hydrogel composed of long fibril could then be accelerated if heating is again applied, confirming the kinetic aspect of GA self-assembly.

The viscoelasticity of GA hydrogels is suggested to derive from a combination of ‘infinite’ rigid helical fibrils, which coexist with shorter aggregates.^8^ These short and long fibrils are structurally identical, only differing by the total number of stacks. Such network of ‘infinite’ fibrils would structure the hydrogel, and their orientation could induce to local nematic ordering towards the shorter fibrils by electrostatic repulsion. The destruction of longer fibrils through shear forces could lead to the shear-thinning behavior of the gel samples, and the formation of short fibrils. The reported self-healing properties of GA gels could come from the reassembly of shorter fibrils into longer ones.

#### Structure

The wide angle X-ray scattering (WAXS) data of lyophilized 24 mM (2 wt%) GA hydrogel indicates that four peaks are formed. These peaks occur at *q* around 2.2, 5.5, 10.3, and 14.3 nm^−1^, corresponding to distances of 2.85, 1.14, 0.61, and 0.44 nm, respectively.^23^ The distance of 2.85 nm is larger than the energy minimized molecular length of GA (1.86 nm), but is less than twice this length, thereby suggesting that the hydrophobic skeleton interact laterally, tilting in a head-to-head way and leaving the hydrophilic headgroups exposed to water (Fig. 2).^23^ This interpretation was later updated by Denk *et al.*,^8^ who suggested that the dimers are more compact, with the hydrophilic groups of the hydrophobic skeleton exposed to water. In this model, the distance of 1.14 nm was related to the length of the headgroup, and the shortest 0.44 nm dimension to the packing of the molecules along the fibril. The diameter of the assembly was measured to be 2.3 nm. In the hydrogel form, the SANS signal of 24 mM (2 wt%) GA in solution forms a slope of −1.64 at relatively low scattering vectors *q*. This slope relates to the form factor of semi-flexible fibrils, and is contrasting to the 5/3 fractal, which is expected from self-avoiding random walks of polymer chains in good solvent. Also, a Porod decay of the scattered intensity decaying in a slope −4 towards high-*q* indicates that the fibrils have a sharp interface with the medium,^23^ so that the hydrophobic–hydrophilic interface is well-defined rather than a gradient with penetration of solvent.

## 4 Perspective and conclusions

The study of self-assembly at the molecular level has unveiled a fascinating world of intricate interactions and complex structures. The exploration of molecules like glycyrrhizic acid (GA) and their unique self-assembly properties not only contributes to our fundamental chemistry and materials science understanding, but also holds promising implications for a wide range of applications. As we look to the future, several key perspectives emerge from the insights gained so far.

### A. Tailored self-assembly for nanomaterials

Understanding the mechanisms of self-assembly, both thermodynamic and kinetic, provides us with a powerful toolkit to engineer novel nanomaterials, either as a self-assembly or template for growth. By carefully tuning parameters such as temperature, concentration, pH, and ionic strength, we can potentially design self-assembling molecules with specific structures and properties tailored for diverse applications, from drug delivery systems to advanced materials. The anisotropic assembly of GA molecules forms one-dimensional (1D) nanofibril nanostructures in water, which, upon increasing concentrations can network into hydrogels, which is an ideal matrix for drug, flavour and aroma encapsulation, as well scaffolds for functional hybrid systems. Here, GA fibrils might play an important role for having a morphology that is easily accessible by simply dissolving this material in water. Helix is a important structural motif for biological systems, such as DNA, collagen, and viruses.^35^ Usually, molecules that can self-assemble into helices either must undergo complex chemical synthesis, or have complex preparation processes with high cost. Here, we show that GA is a single molecule that can self-assemble into these sophisticated hierarchical helical structures simply by being dissolved in water. Furthermore, the fact that these fibrils retain some level of flexibility and develop viscoelasticity after certain length opens opportunities in various fields that could profit from sustainability.

### B. Interplay of forces

The interplay between the hydrophobic effect and hydrogen bonding in driving self-assembly highlights the complexity of GA systems. Future research will likely focus on deciphering the delicate balance between these forces and how it evolves under different formulation conditions, shedding light on the behavior of similar molecules. The GA molecule (and other saponins) have specific features, namely the arrangement and weak separation of hydrophilic and hydrophobic domains, a rigid molecular structure, and their self-assembly is driven by both, the hydrophobic effect and hydrogen binding. This features result in a distinct, complex self-assembly behaviour forming stable fibrils even at low molar concentrations in water.

### C. Challenges in characterization

Despite the clear progress, characterizing the atomic-level structure of micellar aggregates remains a daunting challenge, especially at low concentrations, in which scattering techniques do not generate enough signal. Overcoming this obstacle requires innovative techniques and collaborations across disciplines, bridging the gap between experimental and computational methods to gain a comprehensive understanding of these GA assemblies, probably through modelling. A determination of the micellar structure on an atomic level is nearly impossible at low concentrations, based on experimental methods. These aggregates are possibly highly dynamic, continuously breaking and reforming, with varying structure, which are averaged for any scattering experiment. The measured scattered intensity is often impacted by the interaction effects (structure factor), which are difficult to disentangle from structural information (form factor). Furthermore, this contribution cannot be avoided by dilution, since it changes the shape of the aggregates.

### D. Potential for sustainable solutions

The discovery of self-assembly properties in molecules like GA offers a glimpse into a sustainable future. By leveraging nature-inspired assembly processes, we may develop eco-friendly alternatives to complex and resource-intensive manufacturing methods, reducing our environmental footprint. The discovery that molecules like GA can self-assemble into hierarchically structured fibrils with viscoelastic properties opens doors to sustainable materials and innovative technologies. These fibrils might find applications in fields as diverse as biomedicine, nanotechnology, and environmental science, where their flexibility and stability at low concentrations could be harnessed for various purposes. Intelligent biomaterials, structural framework using the chiral skeleton, (soft-liquid, rigid structure), drug delivery system.^12^ Good use to replace the synthetic low molecular weight hydrogelators.^8^ Due to the pH dependence, the pH solution should be monitored when GA is used as an emulsifier or solubilizer in practical fields. This same pH dependence can be used as triggering, since micelles can be formed at low pH (pH < 5), and disassemble at pH > 7. This feature could be used for drug release applications, for example.

## Author contributions

All authors contributed equally to this work.

## Conflicts of interest

The authors have no conflicts of interest to declare.

## Supplementary Material
